# Effect of Thermal Processing on the Structural and Magnetic Properties of Epitaxial Co_2_FeGe Films

**DOI:** 10.3390/nano14211745

**Published:** 2024-10-30

**Authors:** Andrii Vovk, Dariia Popadiuk, Bogdan Postolnyi, Sergey Bunyaev, Pavel Štrichovanec, José Ángel Pardo, Pedro Antonio Algarabel, Olga Salyuk, Vladislav Korenivski, Gleb N. Kakazei, Vladimir O. Golub, João Pedro Araujo

**Affiliations:** 1 Institute of Physics for Advanced Materials, Nanotechnology and Photonics (IFIMUP), Departamento de Fisica e Astronomia, Faculdade de Ciências, Universidade do Porto, 4169-007 Porto, Portugal; b.postolnyi@fc.up.pt (B.P.); bunyayev@fc.up.pt (S.B.); gleb.kakazei@fc.up.pt (G.N.K.); jearaujo@fc.up.pt (J.P.A.); 2 Nanostructure Physics, Royal Institute of Technology, 10691 Stockholm, Sweden; popadiuk@kth.se (D.P.); vk@kth.se (V.K.); 3 Institute of Magnetism National Academy of Sciences of Ukraine and Ministry of Education and Science of Ukraine, 36-B Vernadsky Blvd., 03142 Kyiv, Ukraine; olga@imag.kiev.ua (O.S.); golub@imag.kiev.ua (V.O.G.); 4 Institute of Spintronics and Quantum Information, Faculty of Physics Adam, Mickiewicz University, 61-712 Poznan, Poland; 5 Department of Nanoelectronics and Surface Modification, Sumy State University, 40007 Sumy, Ukraine; 6 Instituto de Nanociencia y Materiales de Aragón, Universidad de Zaragoza—CSIC, Campus Río Ebro, 50018 Zaragoza, Spain; stricho@unizar.es (P.Š.); jpardo@unizar.es (J.Á.P.); algarabe@unizar.es (P.A.A.); 7 Departamento de Ciencia y Tecnología de Materiales y Fluidos, Universidad de Zaragoza, 50018 Zaragoza, Spain; 8 Instituto de Nanociencia y Materiales de Aragón, Universidad de Zaragoza—CSIC, Campus San Francisco, 50009 Zaragoza, Spain; 9 Departamento de Física de la Materia Condensada, Universidad de Zaragoza, 50009 Zaragoza, Spain

**Keywords:** thin films, Heusler alloys, magnetostatic properties, ferromagnetic resonance

## Abstract

The structure and magnetic properties of epitaxial Heusler alloy films (Co_2_FeGe) deposited on MgO (100) substrates were investigated. Films of 60 nm thickness were prepared by magnetron co-sputtering at different substrate temperatures (T_S_), and those deposited at room temperature were later annealed at various temperatures (T_a_). X-ray diffraction confirmed (001) [110] Co_2_FeGe || (001) [100] MgO epitaxial growth. A slight tetragonal distortion of the film cubic structure was found in all samples due to the tensile stress induced by the mismatch of the lattice parameters between Co_2_FeGe and the substrate. Improved quality of epitaxy and the formation of an atomically ordered L2_1_ structure were observed for films processed at elevated temperatures. The values of magnetization increased with increasing T_S_ and T_a_. Ferromagnetic resonance (FMR) studies revealed 45° in-plane rotation of the easy anisotropy axis direction depending on the degree of the tetragonal distortion. The film annealed at T_a_ = 573 K possesses the minimal FMR linewidth and magnetic damping, while both these parameters increase for another T_S_ and T_a_. Overall, this study underscores the crucial role of thermal treatment in optimizing the magnetic properties of Co_2_FeGe films for potential spintronic and magnonic applications.

## 1. Introduction

Full-Heusler alloys (FHAs) are intermetallic alloys that are characterized by the formula X_2_YZ, where X and Y are transition metals and Z is a s-p element. They are attracting significant interest due to unique physical properties. It was predicted theoretically and shown experimentally that Co_2_FeZ (Z = Al, Ga, Si, Ge) FHAs feature a high Curie temperature, half-metallic properties, large magnetic moment up to 6 μ_B_ per formula unit and a low Gilbert magnetic damping constant α [1,2,3,4,5], making them promising candidates for different spintronic and magnonic applications [6,7]. However, the physical properties of FHAs are highly dependable on their chemical composition, crystal structure and atomic ordering. Thus, special care should be taken during film preparation for specific properties to be achieved. This includes an adequate choice of deposition conditions and post-deposition heat treatments. In some cases, non-monotonic behavior of magnetodynamic properties was observed. Namely, for a low annealing temperature, a decrease in α was found, while further annealing caused a drastic increase in that parameter [5,8]. Importantly, fine-tuning of preparation conditions allows for the formation of Co-based FHA films with half-metallic properties and an extremely low α~0.002 [9,10,11]. Other than deposition conditions, the substrate material itself plays a crucial role in film growth. Substrates like Si covered with native oxide or Corning Glass promote the formation of polycrystalline films [4]. It is worth noting that even in that case, Co_2_FeGe films demonstrate promising structural and magnetic properties, namely L2_1_ atomic ordering and α~0.004 [4]. The single-crystal MgO [100] substrate favors epitaxial conditions with the following relations for growth: (001) [110] Co_2_FeGe || (001) [100] MgO. However, there is a lattice mismatch of ~3.8% because the side diagonal of the MgO unit cell is √2a_MgO_~5.958 Å long [12], while the bulk Co_2_FeGe unit cell is a_Co2FeGe_~5.738 Å [13]. This introduces in-plane tensile strain and thus promotes the tetragonal distortion of the Co_2_FeGe cubic structure. This in-plane strain can be used to control electrical and magnetic properties of any Heusler alloy with a strong coupling between the magnetic order and the lattice [14].

In this work, Co_2_FeGe films were epitaxially grown on MgO (100) substrates with the magnetron co-sputtering technique. The changes in the structure, static and dynamic magnetic properties triggered by different thermal treatments are reported here. It was found that the values of magnetization, direction of the easy axis of the magnetic anisotropy and damping parameter can be altered by adjusting the preparation conditions. Finally, the non-monotonic behavior of the damping parameter with heat treatment observed earlier for certain FHA films was confirmed, and the correlation of the structural features and magnetic properties variations were analyzed.

## 2. Materials and Methods

Epitaxial Co_2_FeGe Heusler alloy films of ~60 nm thickness were deposited onto 10 × 10 mm^2^ single-crystal (001)-oriented MgO substrates (Crystal GmbH, Berlin, Germany) using an Orion-5 sputtering system (AJA International Co., Scituate, MA, USA). The films were prepared with the co-sputtering technique. Two independent direct current magnetrons with 2″ targets were utilized. High-purity (better than 99.99 at. %) Co_2_Fe alloy target was installed in the first magnetron and Ge target in the second. The targets were provided by Testbourn Ltd., Basingstoke, UK. All depositions were made at 3 mTorr of Ar. The deposition rates were fixed as 7.7 nm/min for Co_2_Fe and 5.1 nm/min for Ge. The rates were calculated to obtain Co_2_FeGe film of stoichiometric composition. The films were deposited at different substrates temperatures: room temperature (RT), 573 K and 773 K (the substrate temperature is denoted as T_S_). The films deposited at T_S_ = RT were annealed in-situ in 3 mTorr Ar flow at temperatures (T_a_) 573 K and 773 K for 1 h. For convenience, the samples in this work are marked as follows: S1—deposited at T_S_ = RT; S2—deposited at T_S_ = 573 K; S3—deposited at T_S_ = 773 K; S4—annealed at T_a_ = 573 K; S5—annealed at T_a_ = 773 K. Other technological aspects of film preparation are presented in detail in paper by Vovk et al. [4].

The elemental composition of the films was evaluated by means of energy-dispersive X-ray analysis (FEI Quanta 400FEG field emission scanning electron microscope, EDAX-PEGASUS X4M detector, FEI Co., Hillsboro, OR, USA).

To study the microstructure, the crystal quality and the epitaxy relationships between the films and the substrate, a set of X-ray diffraction (XRD) measurements were carried out using a Rigaku SmartLab high-resolution X-ray diffractometer (Rigaku Co., Tokyo, Japan) and a Cu-Kα radiation source (filtered with a Ge (220) crystal 2-bounce monochromator) operating voltage of 45 kV and current of 200 mA. The measurements were performed in a parallel beam configuration. The XRD experiments included symmetrical and asymmetrical out-of-plane and in-plane scans, rocking curves (ω-scan), and an azimuth ϕ-scan. X-ray reflectivity (XRR) studies were carried out in the same diffractometer to evaluate the thickness, the density, surface oxidation and the surface roughness of the films. The fitting of XRR data was made using LEPTOS software 2.02 (Bruker AXS GmbH, Karlsruhe, Germany). All X-ray studies were carried out in accordance with the recommendations presented in the tutorial [12].

A Quantum Design MPMS SQUID magnetometer (Quantum Design Inc., San Diego, CA, USA) was used to evaluate saturation magnetization (M_S_) and coercive field (H_C_). The measurements were carried out at RT. The magnetic field was applied in the film plane and parallel to the [100] direction of the MgO substrate. The paramagnetic contributions from the substrate and instrumental effects associated with the nanometric scale of the films were analyzed and eliminated following the procedures described in [15,16].

Cavity ferromagnetic resonance (FMR) measurements were carried out at 9.87 GHz (X-band) using a Bruker ELEXSYS-E500 electron spin resonance spectrometer (Bruker AXS GmbH, Karlsruhe, Germany) at room temperature. A two-coordinate sample holder was utilized for measurements of out-of-plane [the polar angle θ_H_ varies from 0 (**H** ǀǀ **n**) to π/2 (**H** ⊥ **n**), the vector **n** being the normal vector to the substrate] and in-plane [the azimuthal angle φ_H_ varies from 0 to 2π, where φ_H_ = 0 corresponds to the [100] direction of the MgO substrate] angular dependencies of the resonance field H_r_. Broadband ferromagnetic resonance (FMR) measurements were performed at RT using a gold coplanar waveguide (CPW) connected to an Anritsu 37247D (Anritsu Co. Inc., Atsugi, Japan) vector network analyzer (VNA). A direct current (DC) magnetic field was applied in the sample plane. The films were positioned face down on the CPW. The transmission coefficient S_21_ was studied as a function of the external magnetic field H over a frequency (f) range from 1 to 20 GHz. The frequency spectra of the complex magnetic susceptibility U(f) were then extracted from the S_21_ raw data using the methods described in [17,18].

## 3. Results and Discussion

### 3.1. Structural and Morphological Characterization

The chemical composition (in atomic percent) for all films was evaluated by EDAX as Co_48_Fe_22_Ge_30_ (±1 at. % error). This confirms high reproducibility during different deposition runs. The composition slightly differs from the targeted stoichiometry, though.

**Figure 1:**
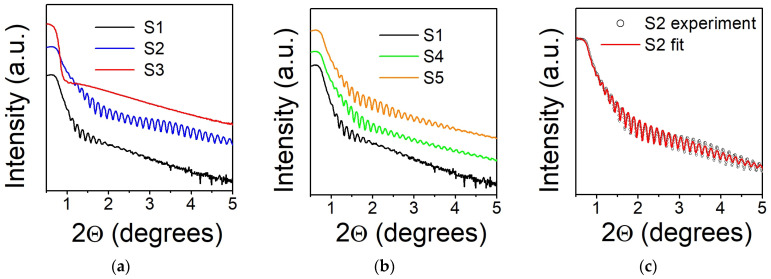
X-ray reflectivity for Co_2_FeGe films on MgO [100] substrates: (**a**) deposited at T_S_ = RT (S1), T_S_ = 573 K (S2), T_S_ = 773 K (S3); (**b**) deposited at T_S_ = RT (S1) and annealed for 1 h at T_a_ = 573 K (S4), T_a_ = 773 K (S5); (**c**) line of best fit of the experimental XRR spectrum for the Sample S2. Fitting parameters are summarized in Table 1.

The XRR patterns of the films are shown in Figure 1.

Samples S1 and S2 show Kiessig fringes in a wide range of scattering angles, suggesting that they have homogeneous thickness and a smooth surface. Sample S3 demonstrates a fast decay of these oscillations, which is a characteristic for a very rough surface (Figure 1a). Meanwhile a periodic-like pattern is preserved for both annealed samples, S4 and S5 (Figure 1b). Fitting of the data (see, for instance, Figure 1c for Sample S2) allows for the estimation of the thickness, the density, and the surface roughness of the films. Because the films were not capped with a protective layer, a native oxide layer was formed on their surface. The presence of this oxide was confirmed in our previous study via transmission electron microscopy for CoFeGe films of different compositions prepared under similar conditions [19]. To improve the fitting of the data, the thicknesses and the densities of the films and native oxide were set as free parameters. The best results for all samples are summarized in Table 1.

The thickness of all Co_2_FeGe films was estimated in the range 57 nm ± 2 nm. This correlates well with the targeted 60 nm value. The thickness of the native oxide layer was evaluated to be about 2 nm. The projected densities of the films are also lower than the Co_2_FeGe alloy bulk values (8.66 g/cm^3^). The lowest one was evaluated for Sample S1. This might be due to the small diffusion rates of atoms during deposition at RT. Deposition at T_S_ = 773 K results in a higher mobility of atoms and a corresponding increase in the films’ density. Also, it should be noted that T_a_ = 773 K is not sufficient to provide effective re-crystallization, with the film density remaining below the bulk value.

The film deposited at T_S_ = RT shows a surface roughness of ~1.4 nm. This value is reduced to ~0.9 nm for T_S_ = 573 K. Similar results were obtained for annealed films with the lowest surface roughness ~0.8 nm for T_a_ = 773 K. On the contrary, deposition at T_S_ = 773 K leads to a dramatic increase in the surface roughness. A similar tendency was observed previously for polycrystalline films deposited on Corning Glass substrates [4]. One of the possible explanations is that the film deposited at T_S_ = 773 K after cooling to RT is subjected to a compressive stress caused by a difference in thermal expansion coefficients between the film and substrate. The relaxation process can generate roughness as atoms near the surface are re-arranging themselves to relieve stress.

**Figure 2:**
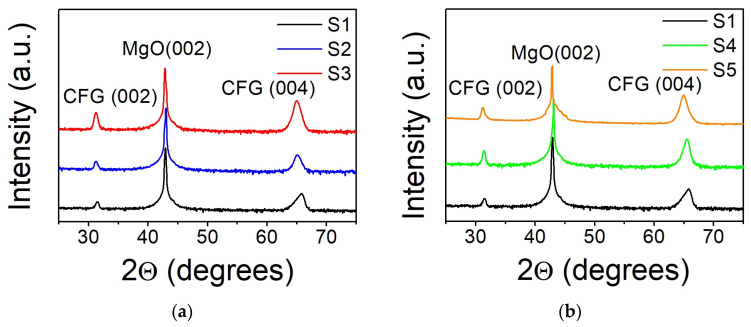
Symmetric 2θ/ω XRD patterns of Co_2_FeGe films on MgO [100] substrates: (**a**) deposited at T_S_ = RT (S1), T_S_ = 573 K (S2), T_S_ = 773 K (S3); (**b**) deposited at T_S_ = RT (S1) and annealed for 1 h at T_a_ = 573 K (S4), T_a_ = 773 K (S5). The acronym CFG is used in the figure to mark the reflections from Co_2_FeGe.

In Figure 2, the symmetric 2θ/ω XRD patterns of Co_2_FeGe films prepared in different conditions are shown. Only (002) and (004) reflections of the Co_2_FeGe films and (002) of the MgO substrate were observed. Peaks from other planes were not detected. This confirms the growth of (001)-oriented films with an out-of-plane epitaxial relationship (001) Co_2_FeGe || (001) MgO. Also, it suggests that at least B2-type atomic ordering for all samples because (002) superlattice reflection is characteristic for that structure [20].

**Figure 3:**
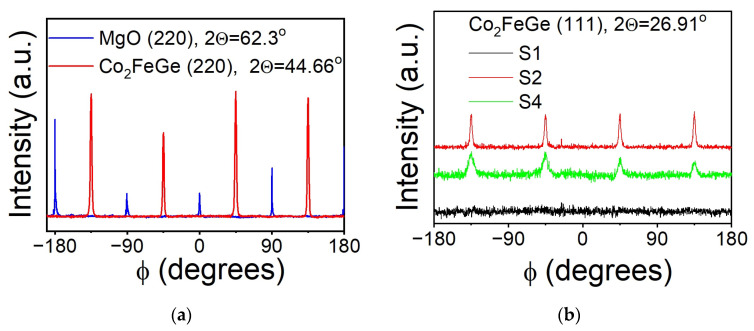
In-plane ϕ-scans for asymmetric (022) reflections from Co_2_FeGe and MgO for Sample S4, deposited at T_S_ = RT and annealed for 1 h at Ta = 573 K (**a**); ϕ-scans for (111) reflection for samples deposited at T_S_ = RT (S1), T_S_ = 573 K (S2), and deposited at T_S_ = RT and annealed for 1 h at T_a_ = 573 K (S4) (**b**).

In-plane film orientation was checked using in-plane ϕ-scans for asymmetric (022) reflections from Co_2_FeGe and MgO. A typical ϕ-scan is presented in Figure 3a for Sample S4. Fourfold symmetry with 90° intervals is clearly seen in the reflections from the Co_2_FeGe film. Those from the MgO substrate are shifted by 45° with respect to the film, which proves the formation of an epitaxial layer with a well-defined in-plane epitaxial relationship [110] Co_2_FeGe || [100] MgO.

Combining asymmetric (220) and symmetric (002) reflections, one can determine the in-plane (*a*) and the out-of-plane (*c*) lattice parameters [21] (see Table 2 for the results).

It is seen that Sample S1 deposited at T_S_ = RT experienced in-plane tensile strain due to the mismatch between the film and substrate lattice parameters. For all the films, the value of the in-plane lattice parameter *a* decreases with an increase in T_S_ and T_a_, while out-of-plane lattice parameter *c* simultaneously increases, keeping the volume of the unit cell almost unchanged. For high values of T_S_ and T_a_, unit cells of the films become almost cubic, manifesting the relaxation of the strain. The estimation of the tetragonal distortion *c*/*a* is also presented in Table 2.

The crystal quality of the films was assessed using rocking curves measured in the vicinity of the (004) reflection. Full width at half maximum (FWHM) values are summarized in Table 2. They are relatively high, especially for Sample S1 (~2.5°). The crystal quality improves with increasing T_S_ and T_a_, although FWHM remains higher than 1°. This is typical for the oxide epitaxy-type growth [12]. The FWHM values in this study are higher than those previously reported for CoFeGe films [19] and for other Heusler alloy films prepared using different deposition techniques [22,23,24]. Relatively high values of FWHM might be due to higher deposition rates compared to those reported in [19]. This can also be related to the absence of a buffer layer for the samples investigated in this work. It is known that a buffer of Cr or Ag could improve conditions for epitaxial growth [24,25,26] due to a smaller lattice mismatch between MgO and Cr (or Ag) compared to Heusler alloys.

The physical properties of Heusler alloys are sensitive to atomic ordering [1]. The fully ordered L2_1_-type structure is the most desirable one. Half metallic ferromagnetic properties were predicted for this structure [1,27]. A defining characteristic of an L2_1_ structure is the presence of superlattice reflections with all odd (hkl) indices, i.e., (111) and (113). One should keep in mind, though, that the same reflexes are also present for partially disordered structures, such as DO_3_, for which Co and Fe or Co and Ge atoms are intermixed on their positions in the crystal lattice [19]. To check the atomic ordering, a set of asymmetrical in-plane ϕ-scans were measured for these specific reflections. It was found that both the (111) and (113) reflections are absent for Sample S1 deposited at T_S_ = RT. Elevated values of T_S_ or T_a_ result in the appearance of both (111) and (113) reflections at 90° intervals of ϕ. In Figure 3b, the ϕ-scans for the (111) reflection of Samples S1, S2, and S4 are shown. It is seen that deposition at T_S_ = 573 K (S2) results in the formation of shaper peaks, while the peaks are broader for the sample annealed at T_a_ = 573 K (S4). These differences might be attributed to the conditions of film crystallization. At an elevated T_S_, films with better atomic ordering are formed due to high migration rates of the atoms. For annealing, recrystallization of atomically disordered films takes place at a slower rate. However, the convenient XRD technique does not allow for a clear differentiation between L2_1_- and DO_3_-ordered phases because of the almost identical atomic scattering factors of the Co, Fe, and Ge atoms. Thus, the conclusion about the presence of a fully ordered L2_1_ phase is not straightforward [1,6], and the estimation of the amount of material in the L2_1_ phase presents a challenge [28]. Moreover, a segregation of the s-p element and the formation of nano- and microregions with composition inhomogeneities were observed both for bulk materials and thin films [29,30,31,32,33]. Nanoscale phase segregation is challenging to detect using macroscopic measurement techniques, like XRD. Nevertheless, the presence of inclusions and local inhomogeneities can affect the relative intensities of XRD reflections. For the films under investigation, an additional factor came into play. Namely, Ge crystalizes in a cubic structure with lattice parameter *a*_Ge_ = 5.6455Å, which belongs to space group *Fd3 $\overset{¯}{m}$*. The strongest peak (111) of Ge overlaps with a weak (111) superlattice reflex of Co_2_FeGe. For Sample S4, the relation between the intensities of the (111) and (220) peaks, *I*_111_/*I*_220_~0.01, is close to the 0.009 value predicted from theoretical calculations [27] (or 0.012 determined from neutron scattering measurements [34]) for the L2_1_-ordered stoichiometric bulk Co_2_FeGe alloy. On the contrary, for the S2, S4, and S5 samples, the relation *I*_111_/*I*_220_ is triple the theoretical value. According to EDAX investigations, the films in this study are slightly enriched with Ge. Thus, the enhanced *I*_111_/*I*_220_ values might indicate partial segregation of Ge and/or the formation of Ge-enriched nanoregions in the films deposited at an elevated T_S_ or annealed at a high T_a_. To prove this assumption, ultra-high-resolution transmission electron microscopy studies might be required because only that technique provides the spatial resolution that is needed. However, an indirect confirmation for the formation of Ge nano-inclusions may be obtained from magnetic measurements too.

### 3.2. Magnetostatic Properties

**Figure 4:**
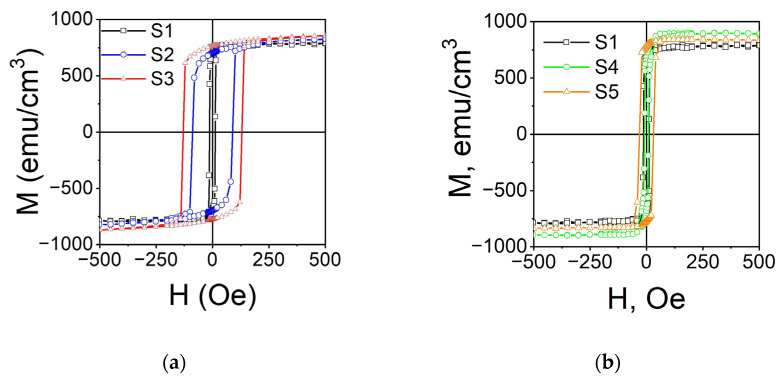
Magnetic hysteresis loops (M vs. H) for Co_2_FeGe films on MgO (100) substrates: (**a**) deposited at T_S_ = RT (S1), T_S_ = 573 K (S2), T_S_ = 773 K (S3); (**b**) deposited at T_S_ = RT (S1) and annealed for 1 h at T_a_ = 573 K (S4), T_a_ = 773 K (S5).

In-plane magnetic hysteresis loops with a magnetic field applied parallel to [100] MgO measured at RT are shown in Figure 4. The values of M_S_ and H_C_ are summarized in Table 3.

The films deposited on MgO substrates show similar saturation magnetization values, within the error limits, but wider hysteresis loops compared to polycrystalline films on Corning Glass substrates, which were prepared in the same conditions [4]. Sample S1 demonstrates the lowest M_S_ among all samples. This can be attributed to the atomically disordered structure of this sample. Both annealing and deposition at elevated temperatures result in an increase in saturation magnetization, which agrees with the structural data that show an improvement in epitaxy and an increase in atomic ordering. The evolution of magnetic properties will be discussed in detail below, in conjunction with the results of magnetodynamic studies.

The values M_S_ obtained for the films under investigation correspond to the magnetic moment ~4–4.8 μ_B_ per formula unit (μ_B_ is Bohr magneton), which is lower than is predicted with the Slater–Pauling rule for the ordered Co_2_FeGe full-Heusler alloy 6 μ_B_/f.u. [1]. The values achieved in this work agree with those that were reported for Co_2_FeGe films previously [35], but they are lower compared to the bulk alloy [13] and foils [36]. The reduction in saturation magnetization can be due to a slight Ge enrichment in the films, along with an atomic disorder and/or tetragonal deformation of the unit cell. Also, the lowering of the estimated value of M_S_ might be associated with the native oxide on the surface of the film.

### 3.3. Magnetodynamic Properties

Let us call to mind the theory of FMR for thin films. The magnetic free energy density of the films F can be presented as

(1) $$F=-H\cdot M+2\pi M_{eff}^{2}m_{z}^{2}-\frac{1}{4}H_{4a}M_{s}(m_{x}^{4}+m_{y}^{4}+m_{z}^{4})-\frac{1}{2}H_{un}(m\cdot e{)}^{2},$$

where **H** is the external magnetic field; **M** is the magnetization vector; M_eff_ is the effective magnetization, $4\pi M_{eff}=4\pi M_{s}-H_{⊥}$; $H_{⊥}$ is the perpendicular uniaxial anisotropy field, which contains all possible contributions—including magnetoelastic H_σ_ [37] and surface roughness induced shape anisotropy H_S_ [38]; H_4a_ is the cubic magnetocrystalline anisotropy field; m_x_, m_y_, and m_z_ are the corresponding projections of the unit vector **m** in the direction of **M**; *x*, *y*, and *z* axes are along Co_2_FeGe crystallographic axes, with *z* being perpendicular to the film plane; and H_un_ is the technologically induced in-plane uniaxial anisotropy with the unit vector **e** showing its direction. In Equation (1), the first term is Zeeman energy, the second is the demagnetizing field, the third is cubic anisotropy energy, and the last one is the in-plane uniaxial anisotropy energy. In spherical coordinate systems with polar and azimuthal angles (φ_H_ and θ_H_), after the minimization of the energy (1), the resonance conditions can be found using a conventional Smit and Beljers formula [39].

(2) $$\omega =\frac{\gamma }{M\sin\theta_{H}}{\left[\frac{\partial^{2}F}{\partial {\theta_{H}}^{2}}\frac{\partial^{2}F}{\partial {\varphi_{H}}^{2}}-{\left(\frac{\partial^{2}F}{\partial \theta_{H}\partial \varphi_{H}}\right)}^{2}\right]}^{\frac{1}{2}}.$$

**Figure 5:**
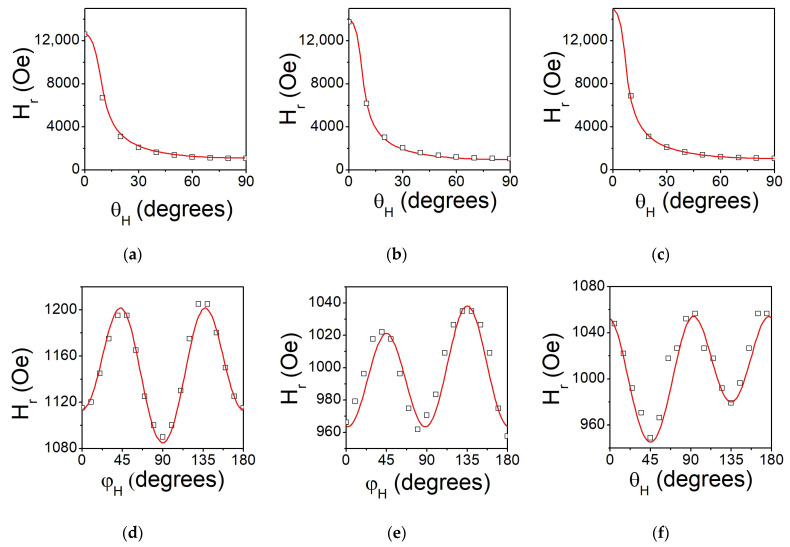
Out-of-plane (top panel) and in-plane (bottom panel) angular dependences of the resonance field H_r_ for samples deposited at T_S_ = RT (S1) (**a**,**d**), deposited at T_S_ = RT and annealed for 1 h at T_a_ = 573 K (S4) (**b**,**e**), and deposited at T_S_ = RT and annealed for 1 h at Ta = 773 K (S5) (**c**,**f**). The lines drawn through the data are the lines of best fit using Equations (1) and (2) for out-of-plane and in-plane, respectively.

Room temperature cavity and broadband FMR measurements were carried out to extract magnetic and magnetodynamic parameters of the investigated films. Firstly, out-of-plane $H_{r}(\theta_{H})$ resonance field angular dependences were measured in cavity to obtain effective magnetization values. The dependencies for the as-deposited (S1) and annealed films (S4 and S5) are shown in Figure 5a–c. The lines of best fit of the experimental data using Equation (2) are presented by solid lines. The extracted magnetic parameters for all investigated films are summarized in Table 3. The film deposited at T_S_ = RT still demonstrates the smallest M_eff_ values, which was attributed to the formation of the film with disordered structure. Both annealing and deposition at elevated temperatures result in an increase in M_eff_, like it was observed in [4] for polycrystalline films of the same composition deposited on Corning Glass substrates. The difference between M_S_ and M_eff_ values can be explained as follows. Tensile strain that appeared at the film–substrate interface results in a tetragonal distortion of the Co_2_FeGe lattice and triggers the appearance of a magnetoelastic component of perpendicular anisotropy. Surface roughness also alters the films’ demagnetizing factors. This causes the corresponding decrease in M_eff_. Meanwhile, the oxide layer on the film’s surface leads to a decrease in the M_S_ calculated from static magnetic measurements but does not affect the values of the M_eff_ determined from FMR. Thus, the values of M_eff_ demonstrate more consistent variations with T_S_ and T_a_. In contrast, the changes in M_S_ with T_S_ and T_a_ obtained from static magnetic measurements for a given set of samples are within the accepted experimental error threshold. This is due to some inaccuracies in measuring the film’s volume and the presence of native oxide on the surface, which affects the evaluations of M_S_.

To study the behavior of in-plane anisotropy, the angular dependencies of the resonance field $H_{r}(\varphi_{H})$ were measured. The dependencies for S1, S4, and S5 samples are presented in Figure 5d–f, and the fitting results are presented in Table 3. $H_{r}(\varphi_{H})$ values for all the films demonstrate almost 90° symmetry, confirming epitaxial growth. A small deviation from perfect four-fold symmetry can be explained by the presence of technologically induced in-plane uniaxial anisotropy H_2a_~10–20 Oe. It is worth noting that for the films with the largest tetragonal lattice distortion (S1, S4), the fourth-order in-plane anisotropy field has negative values (i.e., the easy magnetization direction corresponds to the [110] direction of the Co_2_FeGe lattice)—see Table 2 and Table 3. The largest negative value (H_4a_~−55 Oe) was observed for Sample S1, where this distortion is maximal (*c*/*a* = 0.985). The initial reduction in tetragonal distortion results in a decrease in H_4a_. For Samples S2, S3, and S5 (*c*/*a*~1), the anisotropy field becomes positive (the easy axis coincides with the [100] direction of the Co_2_FeGe lattice, like in the bulk). Such a 45° anisotropy reorientation was previously observed in NiMnGa films and explained in terms of a magnetoelastic interaction [40].

**Figure 6:**
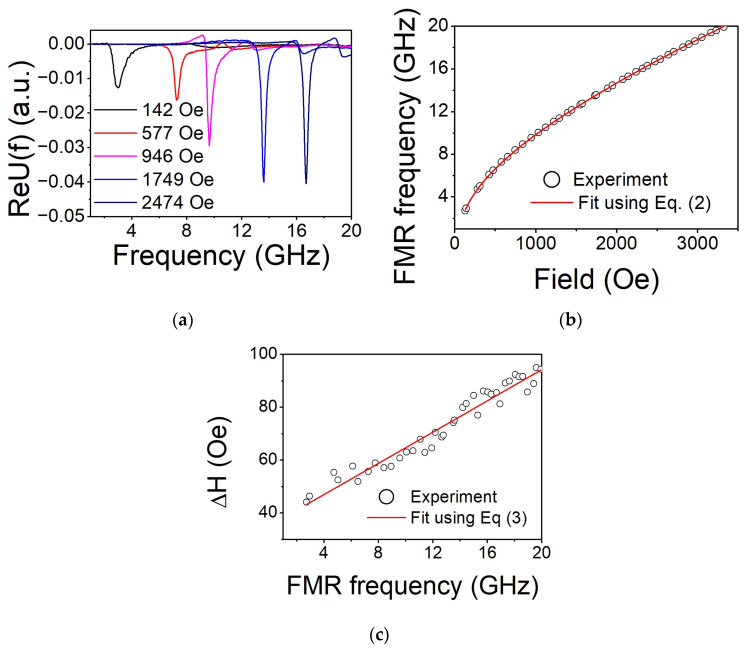
The real part of the U(f) function calculated from the measured complex S2_1_ spectrum at different applied fields (**a**); dependence of the FMR frequency on applied magnetic field obtained using the methodology described in ref. [4]. Experimental points are accompanied by a line of best fit (solid red line) using Equation (2) (**b**); dependence of FMR linewidth ΔH as a function of resonance frequency for Co_2_FeGe S4 sample. Experimental points are accompanied by a line of best fit (solid red line) using Equation (3) (**c**).

To obtain a deeper understanding of the magnetic properties of Co_2_FeGe films, broadband microwave absorption measurements were performed in the frequency domain at various applied magnetic fields (see raw data for S4 in Figure 6a)⁠. The spectra for all the samples contain two resonances, which were identified as uniform FMR precession and first perpendicular standing spin wave modes. The frequencies of the uniform FMR peak were extracted from the raw data, as described in detail in our previous work [4], and fitted using Equation (2). The results of such a fitting for Sample S4 are presented in Figure 6b. The obtained values of M_eff_, H_4a_, and H_2a_ for all the samples are in good agreement with the data collected using cavity FMR.

The FMR resonance linewidth in the most conventional form is described by a formula that contains two terms [17]:

(3) $$∆H=∆H_{0}+\frac{4\pi \alpha f}{\gamma },$$

where the first one, Δ*H*_0_, is a measure of extrinsic broadening related to the film’s quality, and the second one is the intrinsic damping term, with α being the dimensionless Gilbert damping parameter.

Contributions to Δ*H*_0_ are associated with the angular dispersion of crystallite orientations, inhomogeneity of magnetic properties in the material, and two-magnon scattering (which is usually negligible for thick films). Since the first term is frequency-independent and the second one is linearly proportional to *f*, they can be separated and evaluated from broadband FMR measurements using a linear line of best fit (as shown in Figure 6c for Sample S4). The obtained results are summarized in Table 3.

The sample deposited at RT (S1) demonstrates relatively low values of α (~0.009) and Δ*H*_0_. The annealing at T_a_ = 573 K (S4) caused the decrease in α down to 0.0042 and Δ*H*_0_ to 38 Oe. However, depositions at an elevated T_S_ (S2, S3) or annealing at T_a_ = 773 K (S5) lead to a dramatic increase in these parameters. This means that processing at high temperatures might be inappropriate to fabricate epitaxial Co_2_FeGe films for magnonic applications.

It is interesting to note that both α and Δ*H*_0_, as well as H_c_, show non-monotonic dependence on T_a_. One can establish a correlation between the microstructure of the films and the variations of the abovementioned magnetic properties. The film deposited at T_S_ = RT (Sample S1) has a B2-type atomic order, and its crystal structure is tetragonally distorted due to the lattice mismatch-induced in-plane strain. It is characterized by a fine mosaic structure, as revealed by the large FWHM of the rocking curve observed in XRD. Annealing at T_a_ = 573 K (Sample S4) improves the atomic ordering (superlattice reflections for L2_1_-ordered phase appeared), slightly increases the size of the mosaic structure (reduced FWHM value of the rocking curve compared to sample S1), and causes only a partial relaxation of tetragonal distortion. These changes trigger a simultaneous decrease in α, Δ*H*_0_, and H_C_. Annealing at T_a_ = 773 K (Sample S5) promotes better relaxation of the tensile strain and the formation of atomically ordered films with a further increase in mosaic block dimensions (smaller FWHM of rocking curves). However, as was shown above, lattice mismatch-induced strain relaxation leads to a change in the fourth-order magnetic anisotropy alignment. This reorientation might be incomplete or incoherent throughout the different mosaic blocks for given values of T_a_. Additionally, such strain relaxation can be accompanied by the formation of many defects, such as dislocations, stacking faults, etc. It is also possible that processing at elevated temperatures leads to the formation of chemically inhomogeneous areas (e.g., Ge nano-inclusions, which revealed themselves through the abnormally high intensity of the (111) XRD superlattice peak). All these structural defects may cause an increase in α, Δ*H*_0_, and H_C_. Depositions at elevated T_S_ (Samples S2 and S3) stimulate changes in a film’s structure and cause variations in α, Δ*H*_0_, and H_C_, the same way as high-temperature annealing. Thus, for used values of T_S_, only an increase in the abovementioned parameters was observed. It is worth noticing that a similar non-monotonic behavior of α with T_a_ was reported previously for several Heusler alloy films. For example, in ref. [5], Fe_1.5_CoGe films with different thicknesses were studied. It was found that after annealing at T_a_ = 773 K, no visible FMR peak was observed, indicating a degradation of the films’ magnetic properties, whereas at T_a_ = 573 K, a decrease in α was documented. An analogous variation of α with T_a_ was found for Co-based Heusler alloy epitaxial films Co_2_MnAl, Co_2_MnSi, and Co_2_FeSi [8]. In those cases, the initial decrease in α with T_a_ was attributed to the improvements in atomic ordering and the formation of an L2_1_ structure. The subsequent increase in α for T_a_ > 673 K was credited to the diffusion of Cr from a buffer layer to the film and the onset of other unspecified extrinsic effects. In our case, there was no Cr buffer layer; thus, the variation in magnetic properties can be ascribed to the imperfections of the crystal structures of the films.

## 4. Conclusions

The epitaxial growth with the expected (001) [110] Co_2_FeGe || (001) [100] MgO relationships was confirmed. Evaluation of the width of XRD rocking curves suggested that oxide-type epitaxial growth took place. The epitaxial quality improved when T_S_ and T_a_ were increased. The films deposited at T_S_ = RT exhibited some tetragonal distortion associated with in-plane tensile strain caused by the lattice mismatch between Co_2_FeGe and MgO. This distortion was reduced by carrying out processing at elevated temperatures. B2-type atomic ordering of the films was found for T_S_ = RT. For elevated temperatures, additional superlattice reflections (111) and (311) appeared, suggesting the formation of a phase with L2_1_-type atomic ordering or the regions enriched with Ge.

As for the magnetization studies, the lowest M_S_ and M_eff_ values were found for T_S_ = RT. This was attributed to the atomic disorder and the reduced quality of epitaxy. As the deposition temperature increased to 573 K and 773 K, both M_S_ and M_eff_ increased, indicating improved structural, atomic, and magnetic ordering. The film deposited at T_S_ = RT and annealed at T_a_ = 573 K shows increased M_S_ and a reduced coercive field, with respect to the film deposited at T_S_ = RT, confirming better crystal quality. FMR measurements further support these findings, revealing a lower Gilbert damping parameter of α ≈ 0.004 and inhomogeneous broadening of ΔH_0_ ≈ 40 Oe. The films prepared at these conditions are suitable for magnonic applications. On the contrary, for T_a_ = 773 K, a partial degradation of the magnetic properties was detected with FMR via a noticeably larger α and ΔH_0_. This is likely due to the formation of defects and chemical inhomogeneities, such as Ge-enriched nanoregions. Finally, this study showed that the anisotropy axis changes direction with thermal treatment, shifting from the [110] direction of Co_2_FeGe for lower temperatures to the [100] direction for higher temperatures, which is associated with a reduction in tetragonal distortion. These findings provide valuable insights into optimizing the thermal processing conditions for achieving desired structural and magnetic properties in Co_2_FeGe films, enhancing their potential for practical applications.

## Figures and Tables

**Table 1:** Deposition temperature (T_S_); temperature of annealing (T_a_). The film thickness (t), surface roughness (Δt), and density (ρ) were determined from the lines of best fit of the experimental XRR patterns with a chi-squared value below 3 × 10^−2^.

Sample	T_S_, K	T_a_, K	t, nm	Δt, nm	ρ, g/cm^3^
S1	RT	-	55	1.4	8.26
S2	573	-	57	0.9	8.30
S3	773	-	58	5.3	8.50
S4	RT	573	57	1.0	8.30
S5	RT	773	56	0.8	8.30

**Table 2:** Deposition temperature (T_S_), temperature of annealing (T_a_), out-of-plane lattice parameter (*c*), in-plane lattice parameter (*a*), tetragonal distortion (*c*/*a*), volume of the cell (*V*), and full width at half maximum (FWHM) of the (004) rocking curve profile for Co_2_FeGe films. The lattice parameters *a* and *c* were calculated within ±0.002 Å. FWHM was determined from the lines of best fit of the experimental rocking curves with a chi-squared value below 3 × 10^−3^.

Sample	T_S_, K	T_a_, K	*c*, Å	*a*, Å	*c*/*a*	*V*, Å^3^	FWHM, Degrees
S1	RT	-	5.671	5.759	0.985	188.1	2.51
S2	573	-	5.725	5.73	0.999	188.0	1.77
S3	773	-	5.733	5.742	0.999	189.0	0.94
S4	RT	573	5.693	5.759	0.989	188.8	1.85
S5	RT	773	5.735	5.745	0.998	189.3	1.37

**Table 3:** Deposition temperature (T_S_), temperature of annealing (T_a_), saturation magnetization (M_S_) and coercive field (H_C_) determined from SQUID measurements, effective magnetization (M_eff_), the fourth-order magnetic anisotropy field (H_4a_), extrinsic part of resonance linewidth (ΔH_0_), and Gilberts damping parameter (α) determined from FMR measurements. Error margins for M_S_ were estimated from uncertainty of the sample size determination. Error margins for FMR measurements were derived from the fitting procedure.

Sample	T_S_, K	T_a_, K	From SQUID		From FMR			
			M_S_, emu/cm^3^	H_C_, Oe	Meff, emu/cm^3^	H_4a_, Oe	ΔH_0_, Oe	α × 10^3^
S1	RT	-	790 ± 40	14 ± 1	735 ± 20	−55 ± 2	62 ± 1	8.9 ± 0.4
S2	573	-	830 ± 40	90 ± 1	790 ± 20	+27 ± 2	210 ± 3	68 ± 3
S3	773	-	860 ± 40	130 ± 2	900 ± 20	+35 ± 2	640 ± 9	89 ± 4
S4	RT	573	890 ± 45	9 ± 1	840 ± 20	−35 ± 2	38 ± 1	4.2 ± 0.2
S5	RT	773	840 ± 40	30 ± 1	900 ± 20	+48 ± 2	180 ± 3	54 ± 3

## Data Availability

The data presented in this study are available upon request from the corresponding author.
